# 

**Published:** 1907-04

**Authors:** 


### CONTENTS.

ORIGINAL COMMUNICATIONS—
The Signs of Real Death in the Absence of a Doctor. By Wesley E. Taylor, B. S., M. D., Atlanta, Ga........ 1
The Social Evil. By Edgar G. Ballenger, M. D., Lecturer on Genito-Urinary Dseases at the Atlanta School of Medicine, Atlanta, Ga........ 6

EDITORIAL NOTES AND COMMENTS—

Drawn and Undrawn Poulsry........ 14

The Georgia Medical Association........ 16

NEWS AND NOTES........ 21

BOOK REVIEWS........ [illegible]

SELECTIONS AND ABSTRACTS—

Tubercular Adenitis—Treatment by X-Rays........ 27

Paravertebral Triangle of Dulness in Pleural Effusion........ 27

Army Medical Corps Examinations........ 28

Influenza in Relation to the Nervous System........ 29

Causes of Sterility........ 29

Vaccination........ 30

The Choice of Procedure in Loose Kidney........ 30

Chloral Hydrate in Scarlet Fever........ 31

The Salt Free Diet in Chronic Parenchymatous Nephritis........ 32

Hysteria in Children........ 33

"The Practice of Medicine Defined"........ 35

Maternal Impressions and Fetal Malfo rmations........ 36

Health in the Philippines........ 38

Midwives........ 40

On the Sensibflity of Abdominal Organs and the Influence of Injections of Cocaine Upon it........ 42

Robert C. Coffey Reviews the Work of Clark and of Yates Regarding the Mechanical Principles involved in Perineal Drainage........ 44

"The Doctor's Dilemma"........ 47

The Treatment of Eclampsia........ 49

The Paranoia Question........ 52

Surgery of the Spinal Cord........ 56

Influenza........ 60

